# An assessment of genome-editing efficiency of a newly developed Cas9 in *C. elegans*

**DOI:** 10.17912/micropub.biology.000601

**Published:** 2022-07-17

**Authors:** Sakia Ferdousy, Bojun Chen

**Affiliations:** 1 Southern Illinois University Carbondale, Carbondale, IL, USA; 2 BCSIR Laboratories Chattogram, Bangladesh Council of Scientific and Industrial Research, Chattogram, Bangladesh

## Abstract

Minimizing the off-target effects of the programmable genome editing tool CRISPR/Cas9 is critical for its potential therapeutic applications. A recent study reported a new Cas9 variant, SuperFi-Cas9, that dramatically reduces off-target DNA cleavage while retaining on-target DNA cleavage activity in *in vitro* assays. Here we evaluated the genome editing potential of SuperFi-Cas9 by examining its efficiency in mutagenizing targeted genes in the nematode *C. elegans* . We found that gene mutagenesis rates induced by SuperFi-Cas9 through either non-homologous end joining or homology-directed repair were much lower than those by the wild type Cas9, indicating that Super-Cas9 had very low *in vivo* DNA cleavage activity. Our results also suggest that *C. elegans* may serve as an excellent model system for assessing *in vivo* genome-editing efficiency of newly-developed Cas9 variants.

**Figure 1. Comparison of genome editing efficiency between wild type Cas9 and SuperFi-Cas9 in C. elegans:**
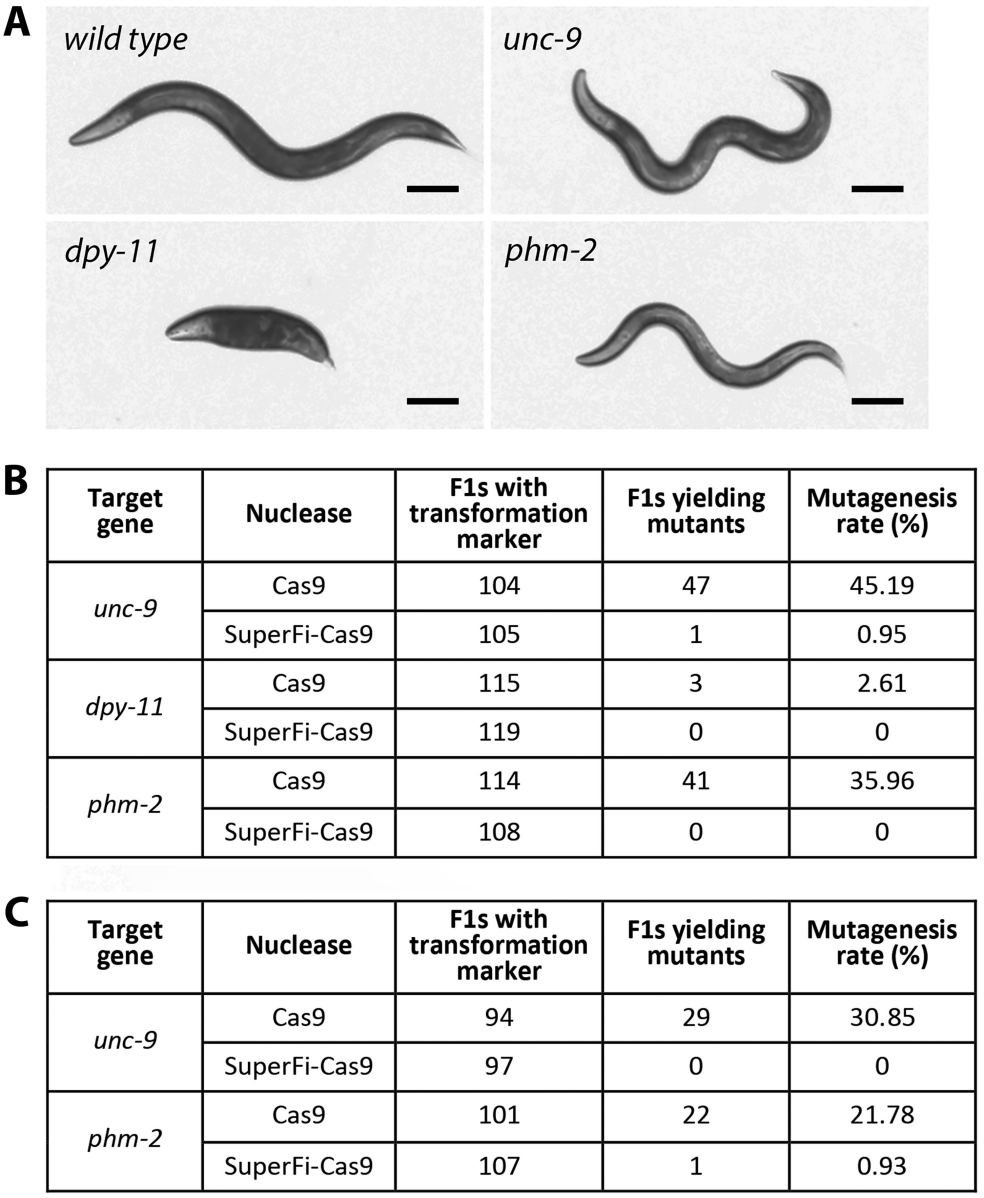
( **A** ) Representative images of wild type and different mutant worms. Worms were grown under identical conditions and the images were taken at young adult stage for all the strains. Scale bar = 100 µm. ( **B** ) Efficiencies of Cas9- and SuperFi-Cas9-mediated mutagenesis through non-homologous end joining. Note that in F1 worms harboring the wild type Cas9, the mutagenesis rates for *unc-9* and *phm-2* were much higher than that for *dpy-11* , which was likely due to the presence of a GG motif at the 3’ end of the protospacer sequences for *unc-9* and *phm-2* but not for *dpy-11* (see methods). This observation is consistent with a previous report showing that a GG motif at the 3’ end of the protospacer sequence dramatically enhances genome editing by CRISPR/Cas9 (Farboud and Meyer, 2015). ( **C** ) Efficiencies of Cas9- and SuperFi-Cas9-mediated mutagenesis through homology-directed repair. Mutagenesis rate = (number of F1s yielding mutants/number of transgenic F1s) x 100.

## Description

One of the important steps towards therapeutic applications of the CRISPR/Cas9-based genome editing technology is to minimize potential off-target DNA cleavage by the Cas9 nuclease. In a recent paper published in Nature (Bravo et al., 2022), the authors engineered a new Cas9 variant, SuperFi-Cas9, based on the structure for Cas9 activation of mismatched DNA determined by kinetics-guided cryo-electron microscopy. When tested *in vitro* , the new Cas9 variant greatly reduced off-target DNA cleavage but maintained comparable on-target DNA cleavage (Bravo *et al.* , 2022). However, it is unclear whether SuperFi-Cas9 may have similar *in vivo* on-target cleavage activity as wild type Cas9. Here we address this question by examining mutagenesis of targeted genes induced by SuperFi-Cas9 and wild type Cas9 in *C. elegans* .

SuperFi-Cas9 was created by mutating seven mismatch-stabilizing residues (Y1010, Y1013, Y1016, V1018, R1019, Q1027, and K1031) of Cas9 to aspartic acid (Bravo *et al.* , 2022). We made the same mutations in Cas9 encoded in an existing plasmid (derived from Addgene plasmid #47549) that also contains an empty sgRNA, which can be modified to target any specific loci in the *C. elegans* genome (Dickinson et al., 2013). We chose three target genes, *unc-9* , *dpy-11* , and *phm-2* , to test the *in vivo* DNA cleavage activity of the Cas9 nucleases. *unc-9* encodes a gap junction protein that plays important roles in electrical coupling between body-wall muscle cells and between neurons (Chen et al., 2007; Shui et al., 2020). Loss-of-function ( *lf* ) mutation of *unc-9* results in distorted body curvature and severely reduced locomotion speed (Chen *et al.* , 2007; Starich et al., 2009). *dpy-11* encodes a membrane-associated thioredoxin-like protein (Ko and Chow, 2002), and mutants of *dpy-11* exhibit dumpy body phenotype in all larval and adult stages (Brenner, 1974). *phm-2* encodes an ortholog of human SAFB (scaffold attachment factor B) and SAFB2 (scaffold attachment factor B2) ( www.wormbase.org ). *phm-2* ( *lf* ) mutants exhibit bacterial avoidance behavior that result in a scrawny body morphology (Kumar *et al.* , 2019). These mutant phenotypes are very easy to distinguish from wild type worms ( **Figure 1A** ).

We injected each CRISPR/Cas9 plasmid into wild type (N2 Bristol) worms, respectively, along with a plasmid encoding mStrawberry under the control of *myo-2* promoter as a transformation marker. Two days after the injection, F1 worms expressing mStrawberry in the pharynx were placed individually on NGM plates and their progeny were screened for mutants based on the phenotypes described above. As shown in **Figure 1B** , mutants were isolated for all three genes from the respective F1 worms carrying plasmids encoding the wild type Cas9. In contrast, only one mutant was recovered for *unc-9* and no mutants were recovered for the other two genes from the progeny of F1 worms harboring SuperFi-Cas9. The extremely low rates of mutagenesis induced by SuperFi-Cas9 indicate that either the new Cas9 variant has very low DNA cleavage activity *in vivo* or it cuts DNA well but for some reasons the double-strand breaks (DSBs) were perfectly repaired. Alternatively, the DSBs caused by SuperFi-Cas9 cannot be repaired through non-homologous end joining (NHEJ), resulting in loss of the edited cells and failure of mutant generation. To address these possibilities, we performed another experiment, in which a corresponding repair oligo was co-injected with the CRISPR/Cas9 plasmids for *unc-9* and *phm-2* , respectively. The repair oligo contains a premature stop codon for the targeted gene so that mutants would be generated through homology-directed repair (HDR) after DNA cleavage. In this experiment, we isolated many mutants for both *unc-9* and *phm-2* from worms expressing wild type Cas9, but only recovered one *phm-2* mutant from worms expressing SuperFi-Cas9 ( **Figure 1C** ), suggesting that SuperFi-Cas9 had very low DNA cleavage activity. Taken together, our results show that the recent reported SuperFi-Cas9 has dramatically reduced genome editing capacity in *C. elegans* compared with the wild type Cas9, and argue that caution should be taken if the new Cas9 variant is to be used for *in vivo* genome editing in other systems.

## Methods

*Worm strains and maintenance:* All worm strains were maintained at 20⁰C on nematode growth media (NGM) seeded with *Escherichia coli* OP50. N2 Bristol was used as wild type.

*Plasmids construction:* pDD162 (P *eft-3* :: *Cas9 + Empty sgRNA* ) was a gift from Bob Goldstein (Addgene plasmid # 47549; http://n2t.net/addgene:47549; RRID:Addgene_47549). This plasmid was modified by inserting a multiple cloning site (MCS) at the empty sgRNA location to facilitate subsequent cloning of protospacer sequence. The seven mutations contained in SuperFi-Cas9 (Bravo *et al.* , 2022) were introduced into the above resultant plasmid ( *cp* 128) using the NEBuilder® HiFi DNA Assembly Master Mix (New England BioLabs, E2621S). The new plasmid ( *cp* 153) harboring SuperFi-Cas9 was fully sequenced to confirm that it does not contain any unwanted mutations. The protospacer sequences for the target genes were then inserted into *cp* 128 and *cp* 153, respectively, using the above same Kit. The protospacer sequences for guide RNAs for *unc-9* , *dpy-11* , and *phm-2* are 5’-ATGAACGGACGAGAATGGG, 5’-TGCTCGGACTTTTCGCCGT, and 5’-GAGAAGCATGTTGCTGAGG. All the plasmids were sequenced before microinjection.

*Generation of mutant worms:* All plasmids for microinjection were purified using Qiagen Spin Miniprep Kit. The CRISPR/Cas9 plasmids (50 ng/µl) were injected into the gonad of young adult worms of wild type hermaphrodite, along with a transformation marker P *myo-2* ::mStrawberry ( *cp* 146, 20 ng/µl). For homology-directed repair experiments, a repair oligo (50 ng/µl) was included in the injection mixture. About 30 worms were injected for each plasmid. The injected worms were placed on NGM plates with OP50 bacteria and kept at 20⁰C for 2 days. For each CRISPR/Cas9 plasmid, about 100 mStrawberry-positive F1 worms were picked to individual plates and allowed to produce progeny for 3 – 4 days at 20⁰C. The plates were then screened for mutant worms based on their phenotypes.

*Imaging:* Images of young adult worms of different strains were taken on NGM plates under a Nikon SMZ18 stereomicroscope equipped with an Allied Vision Mako G-040C camera.

## Reagents

**Table:** 

**Plasmid**	**Genotype**	**Available from**
*cp* 146	P *myo-2::mStrawbery::let-858 3’UTR*	This work
pDD162	P *eft-3::Cas9::tbb-2 3’UTR;* P *U6::empty sgRNA*	Addgene #47549
*cp* 128	P *eft-3::Cas9::tbb-2 3’UTR;* P U6::MCS* + empty sgRNA	This work
*cp* 153	P *eft-3::SuperFi-Cas9::tbb-2 3’UTR;* P U6::MCS* + empty sgRNA	This work
*cp* 154	P *eft-3::Cas9::tbb-2 3’UTR;* P *U6::unc-9 sgRNA*	This work
*cp* 155	P *eft-3::Cas9::tbb-2 3’UTR;* P *U6::dpy-11 sgRNA*	This work
*cp* 75	P *eft-3::Cas9::tbb-2 3’UTR;* P *U6::phm-2 sgRNA*	This work
*cp* 156	P *eft-3::SuperFi-Cas9::tbb-2 3’UTR;* P *U6::unc-9 sgRNA*	This work
*cp* 157	P *eft-3::SuperFi-Cas9::tbb-2 3’UTR;* P *U6::dpy-11 sgRNA*	This work
*cp* 158	P *eft-3::SuperFi-Cas9::tbb-2 3’UTR;* P *U6::phm-2 sgRNA*	This work

*MCS: multiple cloning site

**Table:** 

**Primer**	**Sequence**
*unc-9* repair	TCTCAACGACTTGATGAACGGACGAGAATaaGGGAGGAGTCTGGTCATTTTCCACGTGTA
*phm-2* repair	AGAAACTGGCTCGGGAGAAGCATGTTGCTtaaAGGCGGCGAGCACAATGAGCACTTCCCC
